# Five Years Later—The Impact of the COVID-19 Pandemic on Physical Performance and Cardiometabolic Health Using a Smart Home Gym: An Ecological Case Study

**DOI:** 10.3390/ijerph22050762

**Published:** 2025-05-12

**Authors:** Thalia H. Nguyen, Trent Yamamoto, Dylan Cho, Trevor L. Nguyen, Phillip Goldman, Brett A. Dolezal

**Affiliations:** 1 Airway and UC Fit Digital Health-Exercise Physiology Laboratory, David Geffen School of Medicine, University of California Los Angeles, Los Angeles, CA 90024, USA; tyama@bu.edu (T.Y.); dylanjaec@g.ucla.edu (D.C.); trevornguyen10@g.ucla.edu (T.L.N.); bdolezal@mednet.ucla.edu (B.A.D.); 2 Chobanian and Avedisian School of Medicine, Boston University, Boston, MA 02118, USA; 3 Baylor College of Medicine, Houston, TX 77030, USA; phillip.goldman@bcm.edu

**Keywords:** home-based exercise, smart home gyms, COVID-19, cardiometabolic, fitness

## Abstract

The COVID-19 pandemic has been linked to numerous threats to public health. Of these, physical inactivity became increasingly prevalent, mainly due to the widespread closure of indoor gyms. Home-based exercise alternatives were created as potential solutions, but little research is available validating their efficacy to improve long-term health and fitness. This case study investigated the longitudinal effects of ≥three weekly exercise sessions with a smart home-based fitness platform on anthropometric, fitness, and cardiometabolic measures. Three participants were annually assessed over a five-year period spanning before and after the COVID-19 pandemic. Reductions in body fat percentage occurred simultaneously with increases in fat-free mass. Improvements in physical performance measures, including VO_2_ max and both one-repetition maximum (1-RM) and 85% 1-RM for chest press and squat press, were identified. Cardiometabolic measures also demonstrated notable improvements, as borderline hypertension was reduced along with resting heart rate while resting metabolic rate (RMR) and heart rate variability (HRV) increased. Beyond these metrics, volitional exercise frequency grew without compromising exercise program adherence. Although physical activity on a global scale decreased during the COVID-19 pandemic, the long-term cardiometabolic and fitness benefits observed with this home-based exercise platform highlight its potential to improve health and fitness.

## 1. Introduction

The COVID-19 pandemic incited substantial changes in health, work, and social interaction across the globe. Measures intended to limit transmission, such as social distancing and mask mandates, quickly pivoted to the very movement of people, highlighted by the widespread closure of most indoor and outdoor recreational facilities such as gyms, parks, and beaches. This precautionary, far-reaching shutdown exacerbated a pre-existing, often forgotten pandemic: physical inactivity [1,2]. Reductions in physical activity (PA) were observed globally, attributed primarily to the closure of exercise facilities, prolonged sedentary periods, and increases in screen time [3,4]. Other contributing factors include pre-pandemic PA patterns and varying country-specific regulations intended to mitigate the spread of COVID-19 [5,6,7,8,9]. Sedentarism can have significant health implications, as the link between physical inactivity and chronic disease progression is well-established [10,11]. Ranging from metabolic disorders, cardiovascular disease, and all-cause mortality, the clinical consequences associated with prolonged physical inactivity pose a significant threat to public health [12,13,14]. Effectively treating these comorbidities often requires a nuanced approach, but increasing PA may at the very least represent a partial solution to complex, heterogeneous health issues. The World Health Organization (WHO) estimates that five million deaths could be avoided annually if the global population was more physically active [15].

To this end, the WHO has established general guidelines for recommended PA, which include either 150 min of moderate-intensity physical activity, 75 min of vigorous-intensity physical activity, or an equivalent blend of both per week [15]. While it may have been possible—though unlikely—to meet these recommendations by completing daily household activities (e.g., cleaning, gardening, cooking, etc.) [16], there are several factors that contributed to overarching trends in PA during the COVID-19 pandemic. The availability of cardiovascular and strength training equipment strongly predicted planned PA during this period [17]. Access to home exercise equipment was also connected to key social cognitive determinants of physical activity, including intentions and habits. Beyond accessibility, the use of digital resources, including fitness apps, online tutorials, and daily activity trackers, was also associated with higher levels of PA [18,19,20]. Collectively, these factors played pivotal roles in determining PA, or lack thereof, throughout the pandemic.

Home-based exercise has demonstrated considerable potential as an adjunct intervention for diverse subsets of the population, such as those with chronic obstructive pulmonary disease (COPD) [21] and type 2 diabetes [22,23]. A review on home-based PA prior to and following the COVID-19 pandemic examined a variety of programs designed to improve the functional capabilities of older adults, many of whom are the intended demographic for home-based exercise programs [24]. Comprehensive, multimodal home-based exercise programs have shown the ability to both preserve and improve mobility, balance, strength, and quality of life when consistently adhered to over time [25,26]. For middle-aged adults, low-load resistance exercises performed twice per week for a total of eight weeks have also been shown to reduce depressive symptoms while conferring improvements in vertical jump height, grip strength, and chair stand test performance [27]. Although these findings highlight the benefits of home-based exercise interventions for clinically vulnerable populations, they may be ill-suited for the overall general population. The functional emphasis of these programs, while well intended for their targeted populations, may not offer the rigor or variation of exercises desired by other demographics.

However, coinciding with the onset of COVID-19, nascent technological advances in home-based fitness led to the emergence of connected fitness equipment incorporating virtual training. Targeted towards the general public, such systems have become known as ‘smart home gyms’ or ‘AI-powered home gym equipment’. These digital platforms aimed to improve the user’s physical performance (i.e., fitness) and health (e.g., cardiometabolic) from their own respective residences. Studies indicate that home-based exercise training with virtual trainers can be as effective and cost-efficient as in-person gym training for a range of populations, including both young and older adults, as well as healthy individuals and clinical patients [28,29]. A recent research study investigating a virtual-reality home training platform among elderly individuals demonstrated a notable improvement in health and balance [30]. The ease of access and continuous availability of virtually guided workouts have also led to higher adherence rates in many fitness programs [30,31]. A literature review on smart home gym training identified several benefits pertaining to users’ physiological, psychological, and rehabilitative outcomes [32]. However, it should be noted that the studies reviewed were limited in both number and sample size, especially as it relates to the newer smart home platforms using digital weight technologies. Furthermore, previous research has typically been limited to controlled laboratory settings, highlighting the necessity for free-living exercise training that incorporates real-world behavior, adherence to workout routines, and health-fitness outcomes in everyday environments [33].

Further comprehensive studies are needed to substantiate the efficacy of smart home gyms in real-living settings, especially as it relates to PA patterns and indices of health. Against this background, this case study investigated the effects of smart home gym training benefits on an assortment of fitness and cardiometabolic health outcomes throughout and up to five years following the COVID-19 pandemic in three sedentary young adults. Participants also provided data regarding exercise effort, workout enjoyment, and their evaluation of the smart gym platform. This case study will contribute to the existing body of research demonstrating the effectiveness of exercise training programs that leverage smart home gym platforms within their practical application.

## 2. Material and Methods

### 2.1. Participants

Three apparently healthy (i.e., no disclosed pre-existing comorbidities or health complications) adult Caucasian male participants (average age 25.7 ± 2.0 years, BMI = 27.8) from southern California volunteered for this case study. The inclusion criteria were individuals aged 18–35 with minimal exercise training (<1–2 workouts per month) in the past year. Exclusion criteria included the presence of any significant medical diagnosis such as musculoskeletal, cardiovascular, metabolic, pulmonary, and/or other disorders that limit the ability to exercise or increase the risk of adverse cardiovascular events while exercising. Participants gave written informed consent, and ethical approval was obtained from UCLA (IRB: 11-003190). The research practices were conducted in accordance with the principles outlined in the Declaration of Helsinki. Participants completed a pre-participation physical activity readiness questionnaire (PAR-Q) and an exercise history questionnaire.

### 2.2. Study Design

This case study explored the long-term efficacy of smart home gym training on various fitness, cardiometabolic health outcomes, and subjective measures in three sedentary young adults. The investigation spanned up to five years, with annual assessments conducted throughout and following the COVID-19 pandemic. Dietary intake and macronutrient portions were not controlled. All assessments took place at UC Fit Digital Health–Exercise Physiology Research Laboratory at UCLA, while training was conducted remotely at the respective residences of the study participants. Due to the COVID-19 restrictions on in-laboratory activities during the initial quarantine period (March–December 2020), assessments within this time frame were conducted off-site.

### 2.3. Smart Home Gym

**Figure 1:**
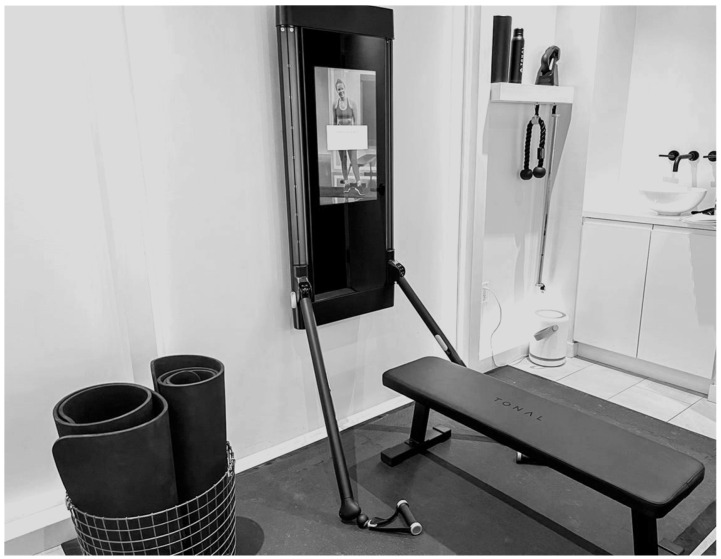
Assembly of a smart home gym (Tonal Gym) with a flat utility bench and accompanying ‘smart’ accessories in a typical residence.

According to the manufacturer, The Tonal Smart Home Gym, developed by Tonal Inc. (San Francisco, CA, USA), serves as a “home-based alternative to traditional training typically conducted at commercial fitness clubs”. It is a vertically wall-mounted multimodal platform with a connected, large touch-enabled flat screen, and two adjustable long arms on each side with handles similar to those found on cable pulley machines powered by patented electromagnets. These are designed to replace traditional weights and provide up to 100 kg of adaptive digital resistance. The adaptive resistance features five dynamic modes designed to customize and enhance users’ workouts similarly to a personal trainer. Tonal uses seventeen sensors to evaluate user form, technique, and speed, providing feedback for self-correction. The ‘smart’ accessories, such as the smart handles and smart bar, also feature a gyroscope motion sensor that connects them to Tonal’s system and tracks repetitions via Bluetooth (Figure 1).

Tonal provides a diverse range of fifteen exercise modalities, encompassing aerobics, resistance training, mobility exercises, and mind–body workouts. Interactive virtual coaching is the central feature of the platform, offering an extensive library of live and on-demand classes with over sixty programs and more than 1000 guided workouts ranging from beginner to advanced levels. Tonal’s classes are instructed by over a dozen different coaches, many of whom teach live sessions in various disciplines including yoga, high-intensity interval training (HIIT), Pilates, strength training, mobility, boot camp, meditation, and more. Tonal offers users the ability to perform over two hundred exercise movements through structured programs, guided workouts, or custom routines. Its database is routinely updated, incorporating innovative programs and guided workouts daily, alongside additional movements introduced every few months. There is a smartphone application that functions similarly to the machine on a smaller screen, aiding in tracking workouts. Furthermore, it includes an on-the-go section with workouts users perform even when not using Tonal Gym.

### 2.4. Five-Year Training Intervention

Participants conducted their training independently from their own residence using Tonal Gym. They had full autonomy over all aspects of their training, provided they engaged in resistance training with Tonal at least thrice per week, ideally incorporating a rest or recovery day between sessions. This requirement aimed to achieve the primary objective of increasing muscle strength and fat-free mass while simultaneously reducing fat mass. Participants were allowed to engage in ad libitum exercise, with or without Tonal, based on their personal preference, provided they adhered to the established guidelines. Weekly compliance was validated based on exercise session data logged by the Tonal machine in each respective home, while other non-Tonal exercise participation was recorded via a digital log. At no point were participants prompted, enticed, or coached to exercise by study research staff. Before the commencement of the study, participants underwent a 2–3 h familiarization session with Tonal. During this session, a comprehensive instructional setup was provided, along with programming and demonstration exercises.

### 2.5. Outcome Measures

Participants were assessed annually over a period of five years, with the beginning of year 0 serving as the baseline in February 2020, conducted one month prior to the COVID-19 lockdown. The subsequent four annual assessments took place within 51 to 53 weeks intervals from the baseline assessment, with the last one completed by March 2024. To ensure accuracy, reliability, and consistency in test administration, all assessments occurred in the same order as listed below, at the same location and time of the day (i.e., early evening to optimize the diurnal effect on performance) by the same investigator. Participants were instructed to ensure that they were well-rested, refrained from exercise for 24 h prior, avoided consuming a large meal and caffeine for at least 4 h before, and arrived fully hydrated and wearing appropriate running shoes.

#### 2.5.1. Cardiometabolic Measures

Anthropometry: Body mass was measured in duplicate on a calibrated medical scale digital BIA scale (accuracy ±0.1 kg) and height was determined using a precision stadiometer (Seca, Hanover, MD, USA; accuracy ±0.01 m). For weight, participants removed all unnecessary clothing, jewelry, glasses, and accessories. For height, participants were instructed to stand as straight as possible with unshod feet flat on the floor.

Body Composition: Body fat percentage, fat mass, and lean body mass was measured using a validated octipolar, multi-frequency, multi-segmental bioelectrical impedance analyzer (R20; InBody Co., Seoul, Republic of Korea) [34]. Participants followed standard BIA guidelines, standing upright with each foot on metallic pads and holding each handle with both hands perpendicular to the floor. The instrument measured resistance and reactance using proprietary algorithms, and body fat percentage, fat mass and fat-free mass were calculated.

Blood Pressure and Resting Heart Rate: Seated blood pressure and resting heart rate were measured in duplicate on the bare left arm after participants rested quietly for a minimum of 10 min using an automated blood pressure system (Omron, Vernon Hills, IL, USA) [35]. Participants were instructed to avoid talking, using their cell phones, or crossing their legs. Cuff size was determined by visual assessment of arm circumference. The same arm was used for all measurements.

Resting Metabolic Rate: A portable metabolic measurement system (COSMED K5, Rome, Italy) was placed next to the participant as they lay in a semi-inclined position in a temperature-controlled (22 C) room with dimmed lighting, absent from noise distraction. Participants donned a standard facemask and head support (Hans Rudolph Inc.^®^, Shawnee, KS, USA) and breathed through a flow sensor that gas analyzer for measures of oxygen consumption (VO_2_) and carbon dioxide production (VCO_2_). Average calorimeter data were calculated for the entire session by analyzing 15-s epochs. The abbreviated Weir formula (EE = 1.44 × (3.9 × [VO_2_ consumed] + 1.1 × [VCO_2_ produced])) was used to determine average resting metabolic rate.

Heart Rate Variability: The participants were fitted with a wrist-worn device and associated smartphone application (Biostrap^®^, Biostrap USA LLC, Los Angeles, CA, USA), to capture a validated photoplethysmography metric, ln rMSSD (i.e., the natural log root mean square of successive R-R interval differences) that measures the vagally mediated HRV response [36]. Using proprietary PPG processing software (Version 1.2.8), the wrist-worn device (Wavelet wristband, Wavelet Health, Mountain View, CA, USA) captured a 60 s reading, producing HRV values with high signal quality [37]. Testing was performed in a comfortable, temperature-controlled (22 °C) room with dim lighting and absent distraction from noise. Subsequently, participants were measured in duplicate while comfortably seated, with the total testing time lasting 10 min.

#### 2.5.2. Fitness Measures

Upper- and Lower-Body Muscle Strength and Endurance: Muscle strength was assessed by the 1-repetition maximum (1-RM) method for bench and squat press exercises. The 1-RM is the highest weight lifted through a full range of motion at the correct speed only completed once. Examiners allowed participants to practice the movement with no load before gradually adding weight and having them perform the first set with 6 to 8 repetitions. After one minute of rest, the load is increased, and the participant performed 3 to 4 repetitions. After 1 min rest, the participant performed 1 to 2 repetitions at a load estimated to be near a maximal effort. Following a final two-minute rest, the participants then attempted their 1-RM. For each 1-RM trial, participants attempted two repetitions. Muscle endurance was then measured as the number of repetitions to failure using 85% of baseline squat and chest press 1-RM values [38].

Lower Body Power: Leg power was estimated using a validated electronic jump mat (Just Jump, Probotics Inc., Huntsville, AL, USA) [39]. Participants stood on a mat with feet at hip-width and then performed a countermovement jump for maximal height. Jump height was recorded with a handheld computer interfaced with the jump mat. Three trials were given with 30 s of rest between trials. The best trial was used to calculate peak leg power using published equations that required jump height and the participant’s body mass. Jump height was determined from “hang-time,” defined as the time (seconds) from the feet leaving the mat to their return and the following equation: Ht = t2 × 1.227 where t is hang-time in seconds, and 1.227 is a constant derived from the acceleration of gravity [40].

Aerobic Performance: Aerobic capacity, VO_2_ max, was determined via gas exchange using an incremental symptom-limited maximal treadmill exercise test. Standard procedures [41], including using individually determined work rate protocols that predict test completion within 8–12 min, were administered. Gas exchange was measured with a portable metabolic measurement system (COSMED K5, Rome, Italy) incorporating a flow sensor and discrete oxygen and carbon dioxide analyzers. Proprietary algorithms show time-aligned flow and gas concentrations breath-by-breath and displayed 8-breath rolling averages for pulmonary minute ventilation (VE), oxygen uptake (VO_2_), and carbon dioxide output (VCO_2_). These metrics were continuously monitored and recorded during 3 min of baseline and throughout exercise and recovery. Heart rate (HR) was assessed via an affixed strap around the chest (Polar Electro Inc.^®^, Kempele, Finland). Maximum oxygen uptake was taken as the highest VO_2_ achieved during a 15 s measurement interval.

Modified Sit-and-Reach Test: The modified sit-and-reach test is a standard measurement tool for evaluating hamstring and lower back flexibility. The test uses a 12-inch sit-and-reach box and the finger-to-box distance as the relative zero point. The participant sat against the wall with their buttocks, shoulders, and head touching it, extended their knees, and placed their feet against the box. A yardstick was positioned on top of the box with the zero end facing the participant. Ensuring that the head and shoulders remained in contact with the wall, the participant extended one hand over the other toward the yardstick, which was adjusted to touch their fingertips. This procedure established the relative zero point for each participant. The participant slowly slid their fingers along the yardstick, recording the farthest point (in cm) they touched. An average of three trials were recorded, with the largest score being used for analysis [42].

#### 2.5.3. Questionnaires

Rating of Perceived Exertion (RPE) Scale: The Borg 6–20 scale was used to measure the rating of perceived exertion after selected training days. The Borg scale allows participants to rate their own perceived level of exertion on a scale from 6 (no exertion at all) to 20 (maximal exertion). RPE scores have been widely used for a half-century, and studies suggest these scores have a high correlation with heart rate [43].

Physical Activity Enjoyment Scale (PACES): The level of enjoyment derived from the workouts was assessed using the Physical Activity Enjoyment Scale. This instrument comprises 16 items, each rated on a scale from 1 (strongly disagree) to 5 (strongly agree), to evaluate the participant’s degree of enjoyment in physical activity. A high overall mean score correlates with an elevated level of enjoyment. PACES results have shown acceptable internal consistency [44].

System Usability Scale: The usability assessment was conducted using the System Usability Scale (SUS), a 10-item questionnaire that has been widely applied to evaluate the usability of various electronic devices and systems. The SUS is a valid and widely used de facto standard for assessing the usability and aims to assess the degree to which a system is fit for its purpose. Its questions assess multiple usability aspects, including ease of use and complexity, and the learning and expertise required to use a system. Each question was graded on a 5-point Likert scale ranging from Strongly agree to Strongly disagree. A composite usability measure is calculated by summing the scores for the odd numbered questions and 5 minus the score for each of the even numbered questions and multiplying the result by 2.5. The resulting SUS measure ranges from 0 to 100, with a measure below 50 considered poor usability, 50–69 considered average usability, 70–84 considered good usability, 85–90 excellent usability, and above 90 best imaginable [45].

### 2.6. Statistical Analysis

This case study’s quantitative aspect included only the means and standard deviations of the outcome measures. Trends are summarized without inferential statistical analysis due to small sample size.

## 3. Results

**Figure 2:**
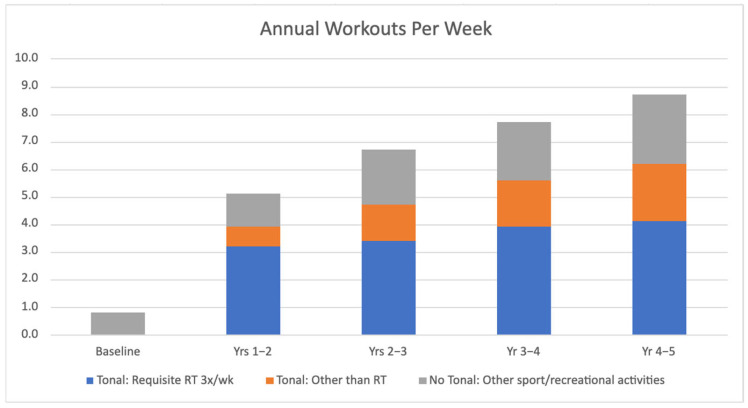
Average annual workouts per week for the requisite thrice-weekly Tonal resistance training (RT), ad libitum workouts using Tonal (e.g., yoga, aerobics, stretching) and other sport/recreational activities not related to Tonal (e.g., jogging, swimming, volleyball).

Three male participants (average age 25.7 ± 2.0 years) were monitored for a duration of 5 years. All participants successfully completed the study without sustaining any injuries or experiencing serious adverse events that would have precluded them from continuing their training. All participants maintained training compliance by completing the requisite minimum three Tonal resistance training sessions per week, each averaging 45 min, throughout the duration of the study. Figure 2 shows the average annual participation rate for the requisite thrice-weekly Tonal resistance training, ad libitum workouts using Tonal (e.g., yoga, aerobics, and stretching) and other sport/recreational activities not related to Tonal (e.g., jogging, swimming, and volleyball).

### 3.1. Anthropometric Measures

**Figure 3:**
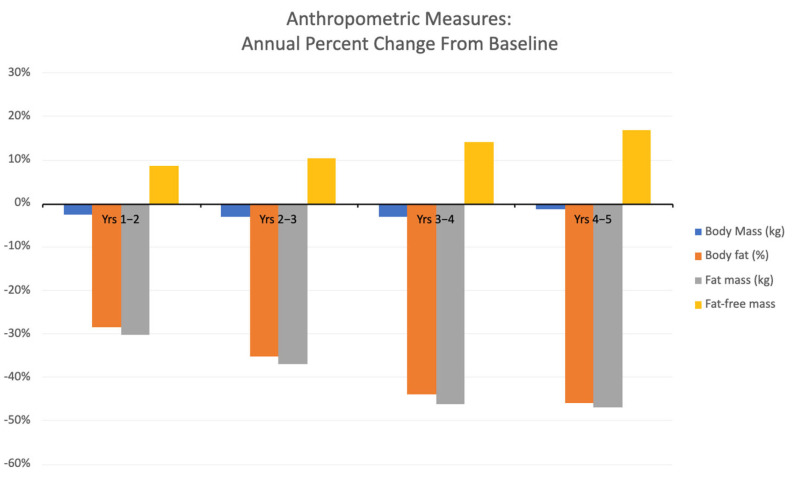
Annual average percentage changes in anthropometric measurements from baseline.

Anthropometric measures are described in Table 1. Body mass decreased slightly after year 1 and then remained stable for the next four years. In contrast, both body fat percentage and fat mass showed a consistent decline from the beginning of the study to its conclusion. Fat-free mass increased steadily over the course of training, culminating in an overall gain of 10 kg, or approximately 17% over five years (Figure 3).

### 3.2. Fitness Outcomes

**Figure 4:**
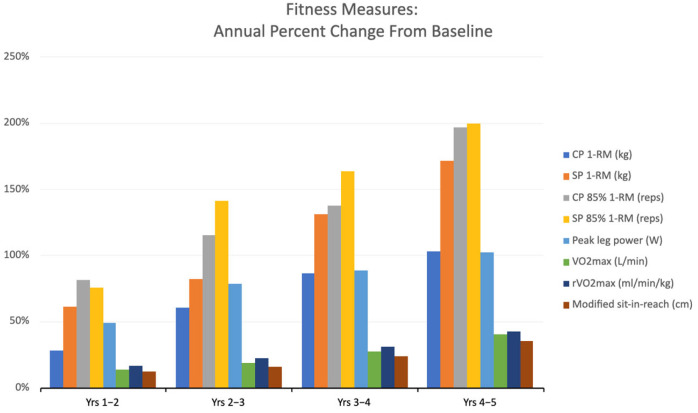
Annual average percentage changes in fitness measurements from baseline.

Fitness measures are described in Table 1. Over the course of 5 years, both upper and lower body muscle strength and endurance increased—chest press 1-RM doubled, and squat 1-RM nearly tripled. Peak lower body power increased twofold, and lower-back/hamstring flexibility improved by one-third by the end of year 5. Aerobic performance progressed from a general classification of ‘below average’ to ‘good’ based on age-and-gender matched norms by the end of training (see Figure 4).

### 3.3. Cardiometabolic Outcomes

**Figure 5:**
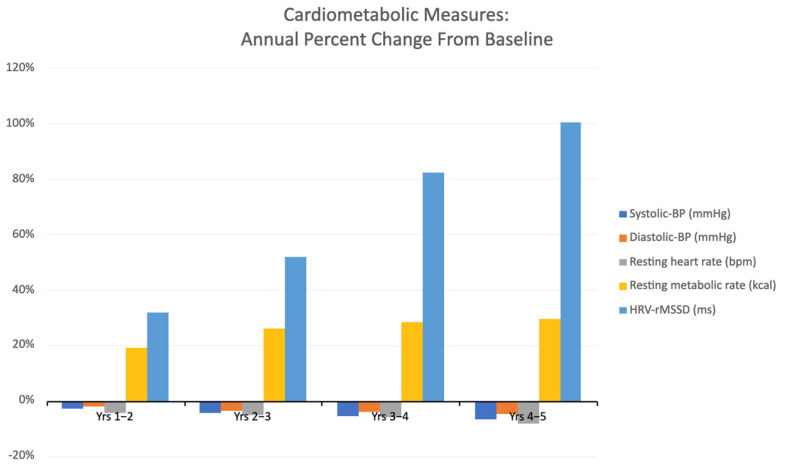
Annual average percentage changes in cardiometabolic measurements from baseline.

Cardiometabolic measures are described in Table 1. Initially, the participants exhibited borderline hypertension (i.e., 129/83), which normalized to a blood pressure of 120/80 by the end of training. By the end of the fifth year, the resting heart rate had progressively decreased by seven beats per minute, from 79 to 72. Additionally, the resting metabolism rose by up to 30%, and the heart rate variability (HRV) doubled (see Figure 5).

### 3.4. Questionnaire Outcomes

During the training, participants rated their Tonal training sessions as “somewhat hard-to-hard” according to RPE criteria with an average RPE score of 14 ± 3.4. Training sessions were described as “enjoyable” according to the PACES scale with an average PACES score of 4.6 ± 0.5. The overall System Usability Scale (SUS) scores were evaluated as “excellent usability” achieving a mean score of 88.5 ± 1.1 on the 100-point Likert scale.

## 4. Discussion

Contrary to the overarching regression of public health associated with the COVID-19 pandemic, this case study demonstrates how persistent home-based exercise can improve health and fitness. Consequently, adopting alternative strategies that incorporate emerging technologies, such as smart home gyms, may prove to be beneficial long-term. Despite the small sample size of three study participants, their five years of dedicated training with Tonal and subsequent improvements in all fitness and cardiometabolic health indices deserve recognition. Overall, a consistent trend emerged across all outcome measures, underscoring the significance of incorporating dynamic training programming, real-time guidance and encouragement—provided in this study through Tonal—as essential components of an effective behavior modification intervention.

The COVID-19 pandemic exacerbated numerous pre-existing health disparities. For instance, the prevalence of obesity, characterized by elevated levels of adiposity, more than doubled in men and women during the pandemic [46]. This increase has been attributed to various factors, including unhealthy eating habits and significantly reduced physical activity. However, participants in the present study experienced a reduction in fat mass along with a concurrent increase in fat-free mass over the five-year period (Figure 3, Table 1). Given that dietary intake was unrestricted for the duration of this study, we posit that the increased frequency of Tonal RT and its alternative exercises, along with other physical activities (Figure 2) contributed to these findings. Resistance training pairs lean weight gain with an equivalent decrease in body fat [47], while aerobic exercise generally is linked to fat mass reduction [48]. This program’s blend of varied exercises may have led to varied effects on body composition, resulting in fat loss. This outcome is not typical for all home-based exercise programs, as discrepancies in body composition trends have been observed in such programs utilized by overweight adults [49].

Another salient trend associated with the COVID-19 pandemic is the increased incidence of cardiovascular disease (CVD), which coincided with waves of excessive mortality [50]. This finding may partly be attributed to the bidirectional relationship between higher levels of adiposity and the risk of CVD-induced mortality [51]. Blood pressure, body mass index, resting heart rate and heart rate variability, all of which were monitored in this study, are well-established risk factors for downstream CVD [52,53,54]. Progressive improvements in these cardiometabolic measures were observed among the participants in this study (Figure 5, Table 1), which may correspond to a decreased risk of CVD. Exposure to increased muscular stress through repeated physical activity has been shown to have advantageous anti-inflammatory effects at the systemic level, with reductions in vascular inflammation and increased vasodilation [55]. The consistent, long-term exercise patterns of these participants likely contributed through a variety of mechanisms, such as those described above, to these cardiometabolic improvements.

From a fitness perspective, notable improvements in all measures of strength and cardiovascular performance were observed over the five-year period. Higher levels of upper and lower-body muscular strength and endurance, combined with aerobic fitness (i.e., VO_2_ max), may be associated with a myriad of health benefits. These factors can confer protective effects against cardiovascular and metabolic disease risk [56]. Moreover, increased muscle fiber recruitment and energy demand resulting from greater muscle mass and strength have been shown to enhance resting metabolic rate, as evidenced in this study, through continuous muscle maintenance and energy expenditure [57]. Additionally, lower body power and flexibility showed gradual improvement over the five-year period. This enhancement has been known to boost mobility, functional strength, and reduce injuries by providing greater stability, muscle coordination, and neuromuscular efficiency [57]. Although it is challenging to determine the singular influence of the smart home gym Tonal on these fitness parameters, considering other concurrent activities, it is reasonable to conclude that the participants’ consistent three to four, 30–40 min workout sessions per week significantly contributed to their fitness outcomes.

At the incipient stage of the pandemic, a surge of publications detailing the physical and mental benefits of physical exercise occurred within the scientific community [58]. This effort was largely coordinated to promote practices that could address the limitations on physical activity imposed by worldwide restrictions. While this investigation was conducted in previously sedentary, but otherwise healthy, young adult participants, the majority of home-based exercise research during this period has been focused on populations with pre-existing health conditions. In a cohort of patients with severe chronic kidney disease, either on hemodialysis or as recipients of kidney transplantation, significant increases in daily average steps, moderate activity, and walking levels were observed in those who underwent a four-month tele-exercise program administered via Zoom [59]. Another small sample of female breast cancer survivors participated in an online home-based exercise intervention, which included two 60 min functional training sessions that were remotely supervised and two sessions of unsupervised aerobic training sessions between 20–30 min each week for 16 weeks [60]. Cardiorespiratory fitness, assessed via the Rockport walking test, along with right arm strength, lower limb strength, and lower limb lean mass were all significantly improved following the intervention.

Indeed, a major focus of existing home-based exercise research during this period has been directed towards individuals who were infected and/or hospitalized by COVID-19. A home-based program, utilizing breathing exercises and stretching, yielded notable improvements in respiratory function (i.e., FEV1 and FEV1/FVC%) in young patients recently recovered from COVID-19 [61]. However, this program produced negligible effects on fatigue. Other programs targeted those more adversely affected by COVID-19, including those hospitalized and/or placed in intensive care. In a 12-week randomized controlled trial, older adults recovering from COVID-19 hospitalization underwent a tele-supervised and home-based training regimen incorporating resistance and aerobic exercises [62]. The therapeutic efficacy of this rehabilitation program was encouraging, as inspiratory and expiratory muscle strength impairment was attenuated while resting SpO_2_ levels were increased. The authors posited that the activation of the respiratory muscles during exercise may have contributed to the observed improvements in respiratory parameters (e.g., maximum inspiratory pressure, maximum expiratory pressure, resting SpO_2_). Another 16-week randomized controlled trial consisting of 50 COVID-19 survivors, who were discharged from the intensive care unit within 5 ± 1 months, examined the effects of a thrice weekly, 60–80 min individualized home-based exercise training program [63]. Following the completion of this exercise program, health-related quality of life, 30-s sit-to-stand performance, and muscle weakness and myalgia were improved in patients who adhered to the program relative to control. While the results of these studies may not be directly comparable to those of the present investigation, the improvements in functional and fitness metrics in these vulnerable populations are generally consistent with the benefits observed in our young, healthy cohort.

Establishing a routine of consistent exercise necessitates a combination of intentional behavioral modification, sustained motivation, and deriving pleasure from the activity [64]. The integration of digital fitness technology into exercise routines has demonstrated its effectiveness as a means for individuals to establish and sustain healthy lifestyle habits. Previous research has indicated that at-home fitness applications enhance adherence to regular exercise by encouraging behavioral modifications and offering short-term health benefits [65]. These findings align with the trend in Figure 2, showing participants gradually increasing their workout frequency, adding an extra Tonal RT session per week by the fifth year. Beyond incorporating additional resistance training and doubling ad libitum workouts using Tonal by the study’s end (e.g., yoga, aerobics, stretching), participants further expanded their exercise repertoire nearly twofold from baseline through other avenues, including various sports and recreational activities. Tonal’s integration of proprietary digital weight technology, artificial intelligence, and expert-led coaching provided participants with a variety of virtual training workouts that they considered “physically challenging”, “emotionally motivating”, and “socially engaging”. The ability to access the equipment from home gave participants the flexibility to choose their workout times and durations. This adaptability has been shown to enhance the long-term frequency of exercise [66].

Tonal’s structured yet flexible approach appeared to support exercise consistency by aligning with psychological principles that influence adherence. Research has identified several factors for maintaining exercise habits that are met by the Tonal platform, including the characteristics of the exercise program, supervision and real-time feedback, data-driven progress monitoring, and enjoyment [67]. The participants’ average RPE score indicated that the workouts provided by Tonal were perceived as challenging but manageable, suggesting an appropriate level of exertion. Additionally, the PACES scores reported by participants indicate that the workouts were considered “highly enjoyable”. As a result, Tonal’s holistic programming, from coaching to structured exercise routines, likely supported their motivation and encouraged long-term engagement. Furthermore, participants rated the platform’s usability as “excellent” on the SUS scale, indicating that Tonal’s intuitive interface contributed significantly to its continued use, given the importance of user-friendliness in regular usage [68].

Various other features of Tonal likely influenced the motivation and consistency of the participants throughout the study, including its use of artificial intelligence. Tonal’s AI-driven weight adjustments and mid-workout adaptations were designed to ensure workouts remained appropriately challenging. Additionally, the platform’s virtual coaching and real-time performance tracking facilitated self-monitoring, goal setting, and progress visualization, which have been associated with improved motivation and exercise adherence [69]. Ultimately, these features helped maintain engagement while addressing common barriers to exercise, such as boredom and stagnation, by continuously adapting to the user’s abilities and goals.

### Limitations, Implications, and Future Directions

While this study has novel implications, it is not immune to the common limitations of case study research, including the reliance on a small sample size lacking a control group. Researcher bias in data interpretation without a control group may be another limitation, as is the reliance on descriptive and not inferential statistics, which ultimately makes it difficult to establish causation. Furthermore, only conducting one annual evaluation may have contributed to further bias, especially given that these may not have accounted for seasonal variations and their effects on participants. The additional influence of unique contextual factors, such as participant motivation levels, access to exercise facilities, variations in instructor expertise, and exercise programming selection, may also not be consistent across different settings. Unrestricted dietary intake may have also played a prominent role in the long-term results of this study, which may also limit the interpretations of both cardiometabolic and anthropometric outcomes. However, the strength of conducting this research in an ecological, free-living environment—where participants engage in exercise under real-world conditions from home—may outweigh some of these limitations by enhancing external validity, capturing authentic behavioral patterns, and improving the applicability of findings to everyday practice. Future research should be directed towards examining the long-term effects of home-based exercise using this platform in different populations with larger sample sizes, in addition to doing so with more frequent annual evaluations and regulation of dietary intake.

## 5. Conclusions

Home-based exercise was adopted as an alternative to traditional exercise facilities as a result of widespread closures during the COVID-19 pandemic. In this study’s small cohort, the Tonal smart home gym effectively addressed common exercise barriers, including time constraints, gym accessibility, monotonous and generic workout routines, and plateauing progress. Long-term exercise adherence, coupled with enhanced cardiometabolic health and fitness outcomes, was observed in this ecological intervention in each respective residence. For individuals previously leading sedentary lifestyles who have encountered difficulties with traditional exercise methods, Tonal may prove to be a more effective catalyst for initiating and maintaining long-term fitness.

## Figures and Tables

**Table 1:** Anthropometric, fitness, and cardiometabolic measures annually for five years.

Measures	N = 3				
	Baseline−Yr 0	Yrs 1–2	Yrs 2–3	Yrs 3–4	Yrs 4–5
Anthropometric					
Height (cm)	175 (2.1)	-	-	-	-
Body mass (kg)	84.1 (2.2)	81.9 (7.1)	81.5 (6.3)	81.5 (5.4)	82.9 (7.0)
Body fat (%)	28.7 (2.9)	20.4 (2.2)	18.6 (2.8)	16.1 (2.5)	15.5 (2.2)
Fat mass (kg)	24.1 (3.4)	16.8 (3.4)	15.2 (2.8)	13.0 (2.4)	12.8 (2.2)
Fat-free mass (kg)	60.0 (2.4)	65.2 (2.8)	66.3 (1.2)	68.5 (2.8)	70.1 (2.8)
Fitness					
CP 1-RM (kg)	51.4 (7.5)	65.7 (9.0)	82.5 (7.8)	95.8 (8.1)	104.5 (9.8)
SP 1-RM (kg)	54.7 (9.7)	88.2 (17.2)	99.8 (13.5)	126.5 (12.8)	148.5 (9.8)
CP 85% 1-RM (reps)	3.2 (0.8)	5.8 (1.9)	6.9 (1.7)	7.6 (2.3)	9.5 (1.5)
SP 85% 1-RM (reps)	4.1 (2.6)	7.2 (2.1)	9.9 (3.4)	10.8 (4.8)	12.3 (4.2)
Leg power_peak_ (W)	1655 (175)	2466 (331)	2950 (187)	3122 (220)	3345 (430)
VO_2_ max (L/min)	2.83 (0.79)	3.21 (0.52)	3.36 (0.49)	3.60 (1.02)	3.98 (0.88)
rVO_2_ max (mL/min/kg)	33.65 (3.52)	39.19 (2.54)	41.22 (2.10)	44.17 (2.4)	48.01 (4.76)
* Sit-in-reach (cm)	34.3 (4.1)	38.5 (5.1)	39.8 (3.1)	42.4 (6.7)	46.5 (6.8)
Cardiometabolic					
Systolic-BP (mmHg)	128.9 (2.7)	125.2 (3.1)	123.5 (1.4)	122.0 (3.6)	120.4 (4.3)
Diastolic-BP (mmHg)	83.4 (2.3)	81.7 (2.5)	80.4 (1.6)	80.1 (2.4)	79.5 (2.3)
Resting Heart Rate (bpm)	78.5 (2.3)	75.1 (2.0)	74.4 (1.6)	74.0 (2.9)	72.2 (1.8)
RMR (kcal)	1785 (199)	2130 (125)	2250 (185)	2290 (225)	2310 (350)
HRV-rMSSD (ms)	32.8 (2.7)	43.3 (5.6)	49.8 (4.5)	59.8 (6.8)	65.8 (6.5)

Values are mean (SD). CP = chest press; SP = squat press; 1-RM = 1-repetition maximum; VO_2_ max = maximum oxygen uptake; rVO_2_ max = maximum oxygen uptake normalized by body mass; BP = blood pressure; RMR = resting metabolic rate; HRV-rMSSD = heart rate variability-root mean square of successive differences between normal heartbeats; * Modified sit-and-reach test.

## Data Availability

The datasets used and/or analyzed during the current study are available from the corresponding author upon reasonable request.

## References

[1] Gomez D., Neufeld E.V., Hicks J.W., Dolezal B.A. (2021). COVID-19 Lockdowns: Exacerbating the Silent Pandemic. Int. J. Exerc. Sci..

[2] Fuzeki E., Groneberg D.A., Banzer W. (2020). Physical activity during COVID-19 induced lockdown: Recommendations. J. Occup. Med. Toxicol..

[3] Ammar A., Brach M., Trabelsi K., Chtourou H., Boukhris O., Masmoudi L., Bouaziz B., Bentlage E., How D., Ahmed M. (2020). Effects of COVID-19 Home Confinement on Eating Behaviour and Physical Activity: Results of the ECLB-COVID19 International Online Survey. Nutrients.

[4] Smith L., Jacob L., Trott M., Yakkundi A., Butler L., Barnett Y., Armstrong N.C., McDermott D., Schuch F., Meyer J. (2020). The association between screen time and mental health during COVID-19: A cross sectional study. Psychiatry Res..

[5] Valeriani F., Protano C., De Giorgi A., Mazzeo E., Liguori G., Romano Spica V., Vitali M., Gallè F. (2023). Analysing features of home-based workout during COVID-19 pandemic: A systematic review. Public Health.

[6] Mutz M., Gerke M. (2020). Sport and exercise in times of self-quarantine: How Germans changed their behaviour at the beginning of the Covid-19 pandemic. Int. Rev. Sociol. Sport.

[7] DeJong A.F., Fish P.N., Hertel J. (2021). Running behaviors, motivations, and injury risk during the COVID-19 pandemic: A survey of 1147 runners. PLoS ONE.

[8] Gjestvang C., Tangen E.M., Haakstad L.A.H. (2022). The Coronavirus pandemic and closed fitness clubs negatively affected members exercise habits. Front. Sports Act. Living.

[9] Symons M., Cunha C.M., Poels K., Vandebosch H., Dens N., Cutello C.A. (2021). Physical Activity during the First Lockdown of the COVID-19 Pandemic: Investigating the Reliance on Digital Technologies, Perceived Benefits, Barriers and the Impact of Affect. Int. J. Environ. Res. Public Health.

[10] Anderson E., Durstine J.L. (2019). Physical activity, exercise, and chronic diseases: A brief review. Sports Med. Health Sci..

[11] Booth F.W., Roberts C.K., Laye M.J. (2014). Lack of exercise is a major cause of chronic disease. Compr. Physiol..

[12] Bourke E., Rawstorn J., Maddison R., Blakely T. (2024). The effects of physical inactivity on other risk factors for chronic disease: A systematic review of reviews. Prev. Med. Rep..

[13] Moxley E., Webber-Ritchey K.J., Hayman L.L. (2022). Global impact of physical inactivity and implications for public health nursing. Public Health Nurs..

[14] Park J.H., Moon J.H., Kim H.J., Kong M.H., Oh Y.H. (2020). Sedentary Lifestyle: Overview of Updated Evidence of Potential Health Risks. Korean J. Fam. Med..

[15] World Health Organization (2024). Physical Activity. https://www.who.int/news-room/fact-sheets/detail/physical-activity.

[16] Carvalho V.O., Gois C.G. (2020). COVID-19 pandemic and home-based physical activity. J. Allergy Clin. Immunol..

[17] Kaushal N., Keith N., Aguiñaga S., Hagger M.S. (2020). Social Cognition and Socioecological Predictors of Home-Based Physical Activity Intentions, Planning, and Habits during the COVID-19 Pandemic. Behav. Sci..

[18] Mutz M., Müller J., Reimers A.K. (2021). Use of Digital Media for Home-Based Sports Activities during the COVID-19 Pandemic: Results from the German SPOVID Survey. Int. J. Environ. Res. Public Health.

[19] Schneider F., Runer A., Burkert F., Aspang J., Reider S., Schneider H., Pocecco E. (2022). Digital Workout Versus Team Training: The Impact of the COVID-19 Pandemic on Athletes. Sports Med. Int. Open.

[20] Parker K., Uddin R., Ridgers N.D., Brown H., Veitch J., Salmon J., Timperio A., Sahlqvist S., Cassar S., Toffoletti K. (2021). The Use of Digital Platforms for Adults’ and Adolescents’ Physical Activity During the COVID-19 Pandemic (Our Life at Home): Survey Study. J. Med. Internet Res..

[21] Jung T., Moorhouse N., Shi X., Amin M.F. (2020). A Virtual Reality-Supported Intervention for Pulmonary Rehabilitation of Patients With Chronic Obstructive Pulmonary Disease: Mixed Methods Study. J. Med. Internet Res..

[22] Guadalupe-Grau A., López-Torres O., Martos-Bermúdez Á., González-Gross M. (2020). Home-based training strategy to maintain muscle function in older adults with diabetes during COVID-19 confinement. J. Diabetes.

[23] Ooi T.C., Ludin A.F.M., Loke S.C., Singh M.A.F., Wong T.W., Vytialingam N., Abdullah M.M.J.A., Ng O.C., Bahar N., Zainudin N. (2021). A 16-Week Home-Based Progressive Resistance Tube Training Among Older Adults With Type-2 Diabetes Mellitus: Effect on Glycemic Control. Gerontol. Geriatr. Med..

[24] Maio M.D., Bratta C., Iannaccone A., Castellani L., Foster C., Cortis C., Fusco A. (2022). Home-Based Physical Activity as a Healthy Aging Booster before and during COVID-19 Outbreak. Int. J. Environ. Res. Public Health.

[25] Sadeghi H., Jehu D.A., Daneshjoo A., Shakoor E., Razeghi M., Amani A., Hakim M.N., Yusof A. (2021). Effects of 8 Weeks of Balance Training, Virtual Reality Training, and Combined Exercise on Lower Limb Muscle Strength, Balance, and Functional Mobility Among Older Men: A Randomized Controlled Trial. Sports Health.

[26] Mañas A., Gómez-Redondo P., Valenzuela P.L., Morales J.S., Lucía A., Ara I. (2021). Unsupervised home-based resistance training for community-dwelling older adults: A systematic review and meta-analysis of randomized controlled trials. Ageing Res. Rev..

[27] Kikuchi N., Ohta T., Hashimoto Y., Mochizuki Y., Saito M., Kozuma A., Deguchi M., Inoguchi T., Shinogi M., Homma H. (2023). Effect of Online Home-Based Resistance Exercise Training on Physical Fitness, Depression, Stress, and Well-Being in Middle-Aged Persons: A Pilot Study. Int. J. Environ. Res. Public Health.

[28] Peterlin J., Dimovski V., Colnar S., Blažica B., Kejžar A. (2024). Older adults’ perceptions of online physical exercise management. Front. Public Health.

[29] Liu J., Liu Y., Chen V., Chee W., Im E.O. (2024). Feasibility and acceptability of a home-based virtual group exercise program in global Asian adult population: Baseline characteristics of a cohort study. Medicine.

[30] Cho G.H., Hwangbo G., Shin H.S. (2014). The Effects of Virtual Reality-based Balance Training on Balance of the Elderly. J. Phys. Ther. Sci..

[31] Annesi J.J., Mazas J. (1997). Effects of virtual reality-enhanced exercise equipment on adherence and exercise-induced feeling states. Percept. Mot. Skills.

[32] Qian J., McDonough D.J., Gao Z. (2020). The Effectiveness of Virtual Reality Exercise on Individual’s Physiological, Psychological and Rehabilitative Outcomes: A Systematic Review. Int. J. Environ. Res. Public Health.

[33] Argent R., Daly A., Caulfield B. (2018). Patient Involvement With Home-Based Exercise Programs: Can Connected Health Interventions Influence Adherence?. JMIR Mhealth Uhealth.

[34] Dolezal B.A., Lau M., Abrazado M., Storer T.W., Cooper C.B. (2013). Validity of two commercial grade bioelectrical impedance analyzers for measurement of body fat percentage. J. Exerc. Physiol. Online.

[35] Pickering T.G., Hall J.E., Appel L.J., Falkner B.E., Graves J.W., Hill M.N., Jones D.H., Kurtz T., Sheps S.G., Roccella E.J. (2005). Recommendations for blood pressure measurement in humans: An AHA scientific statement from the Council on High Blood Pressure Research Professional and Public Education Subcommittee. J. Clin. Hypertens..

[36] Hu J., Browne J., Baum J., Robinson A., Arnold M., Reid S., Neufeld E., Dolezal B.A. (2020). Lower limb graduated compression garments modulate autonomic nervous system and improve post-training recovery measured via heart rate variability. Int. J. Exerc. Sci..

[37] Dur O., Rhoades C., Ng M.S., Elsayed R., Mourik R.V., Majmudar M.D. (2018). Design rationale and performance evaluation of the wavelet health wristband: Benchtop validation of a wrist-worn physiological signal recorder. JMIR Mhealth Uhealth.

[38] Baechle T., Earle R. (2008). Essentials of Strength Training and Conditioning/National Strength and Conditioning Association.

[39] Leard J.S., Cirillo M.A., Katsnelson E., Kimiatek D.A., Miller T.W., Trebincevic K., Garbalosa J.C. (2007). Validity of two alternative systems for measuring vertical jump height. J. Strength Cond. Res..

[40] Harman E.A., Rosenstein M.T., Frykman P.N., Rosenstein R.M., Kraemer W.J. (1991). Estimation of human power output from vertical jump. J. Strength Cond. Res..

[41] Cooper C.B., Storer T.W. (2001). Exercise Testing and Interpretation: A Practical Approach.

[42] Thompson W.R. (2010). ACSM’s Guidelines for Exercise Testing and Prescription.

[43] Williams N. (2017). The Borg Rating of Perceived Exertion (RPE) scale. Occup. Med..

[44] Kendzierski D., DeCarlo K.J. (1991). Physical Activity Enjoyment Scale: Two Validation Studies. J. Sport. Exerc. Psychol..

[45] Brooke J. (1996). Usability Evaluation in Industry. SUS-A Quick and Dirty Usability Scale. https://digital.ahrq.gov/sites/default/files/docs/survey/systemusabilityscale%2528sus%2529_comp%255B1%255D.pdfwebcite.

[46] Nour T.Y., Altintaş K.H. (2023). Effect of the COVID-19 pandemic on obesity and it is risk factors: A systematic review. BMC Public Health.

[47] Westcott W.L. (2012). Resistance Training is Medicine: Effects of Strength Training on Health. Curr. Sports Med. Rep..

[48] Drenowatz C., Hand G.A., Sagner M., Shook R.P., Burgess S., Blair S.N. (2015). The Prospective Association between Different Types of Exercise and Body Composition. Med. Sci. Sports Exerc..

[49] Power S., Rowley N., Flynn D., Duncan M., Broom D. (2022). Home-based exercise for adults with overweight or obesity: A rapid review. Obes. Res. Clin. Pract..

[50] Han L., Zhao S., Li S., Gu S., Deng X., Yang L., Ran J. (2023). Excess cardiovascular mortality across multiple COVID-19 waves in the United States from March 2020 to March 2022. Nat. Cardiovasc. Res..

[51] Kim M.S., Kim W.J., Khera A.V., Kim J.Y., Yon D.K., Lee S.W., Shin J.I., Won H.H. (2021). Association between adiposity and cardiovascular outcomes: An umbrella review and meta-analysis of observational and Mendelian randomization studies. Eur. Heart J..

[52] Fuchs F.D., Whelton P.K. (2020). High Blood Pressure and Cardiovascular Disease. Hypertension.

[53] Khan S.S., Ning H., Wilkins J.T., Allen N., Carnethon M., Berry J.D., Sweis R.N., Lloyd-Jones D.M. (2018). Association of Body Mass Index With Lifetime Risk of Cardiovascular Disease and Compression of Morbidity. JAMA Cardiol..

[54] Fang S.C., Wu Y.L., Tsai P.S. (2020). Heart Rate Variability and Risk of All-Cause Death and Cardiovascular Events in Patients With Cardiovascular Disease: A Meta-Analysis of Cohort Studies. Biol. Res. Nurs..

[55] Green D.J., Smith K.J. (2018). Effects of Exercise on Vascular Function, Structure, and Health in Humans. Cold Spring Harb. Perspect. Med..

[56] Artero E.G., Lee D., Lavie C.J., España-Romero V., Sui X., Church T.S., Blair S.N. (2013). Effects of Muscular Strength on Cardiovascular Risk Factors and Prognosis. J. Cardiopulm. Rehabil. Prev..

[57] Vancini R.L., Andrade M.S., Viana R.B., Nikolaidis P.T., Knechtle B., Campanharo C.R.V., de Almeida A.A., Gentil P., de Lira C.A.B. (2021). Physical exercise and COVID-19 pandemic in PubMed: Two months of dynamics and one year of original scientific production. Sports Med. Health Sci..

[58] Michou V., Nikodimopoulou M., Liakopoulos V., Anifanti M., Tsamos G., Vasdeki D., Panayiotou G., Mameletzi D., Deligiannis A., Kouidi E. (2024). Home-based tele-exercise training and physical activity during the COVID-19 pandemic in chronic kidney disease patients. J. Nephrol..

[59] Sagarra-Romero L., Butragueño J., Gomez-Bruton A., Lozano-Berges G., Vicente-Rodríguez G., Morales J.S. (2022). Effects of an online home-based exercise intervention on breast cancer survivors during COVID-19 lockdown: A feasibility study. Support. Care Cancer.

[60] Jeong C., Nam M., Lee D., Hong J., Yu J., Kim J., Kim S., Nam Y. (2024). Randomized Controlled Trial on the Effects of Home-Based Breathing Exercises on Respiratory Function and Fatigue in COVID-19-Cured Young Patients. Healthcare.

[61] Amaral V.T.D., Viana A.A., Heubel A.D., Linares S.N., Martinelli B., Witzler P.H.C., Oliveira G., Zanini G.D.S., Silva A.B., Mendes R.G. (2022). Cardiovascular, Respiratory, and Functional Effects of Home-Based Exercise Training after COVID-19 Hospitalization. Med. Sci. Sports Exerc..

[62] Longobardi I., Goessler K., de Oliveira Júnior G.N., do Prado D., Santos J.V.P., Meletti M.M., Oliveira de Andrade D.C., Gil S., Boza J., Lima F. (2023). Effects of a 16-week home-based exercise training programme on health-related quality of life, functional capacity, and persistent symptoms in survivors of severe/critical COVID-19: A randomised controlled trial. Br. J. Sports Med..

[63] Hughes D.C., Ellefsen S., Baar K. (2018). Adaptations to Endurance and Strength Training. Cold Spring Harb. Perspect. Med..

[64] Rhodes R.E., Sui W. (2021). Physical Activity Maintenance: A Critical Narrative Review and Directions for Future Research. Front. Psychol..

[65] Glynn L.G., Hayes P.S., Casey M., Glynn F., Alvarez-Iglesias A., Newell J., ÓLaighin G., Heaney D., O’Donnell M., Murphy A.W. (2014). Effectiveness of a smartphone application to promote physical activity in primary care: The SMART MOVE randomised controlled trial. Br. J. Gen. Pract..

[66] Jakicic J.M., Winters C., Lang W., Wing R.R. (1999). Effects of Intermittent Exercise and Use of Home Exercise Equipment on Adherence, Weight Loss, and Fitness in Overweight Women: A Randomized Trial. JAMA.

[67] Collado-Mateo D., Lavín-Pérez A.M., Peñacoba C., Del Coso J., Leyton-Román M., Luque-Casado A., Gasque P., Fernández-del-Olmo M.Á., Amado-Alonso D. (2021). Key Factors Associated with Adherence to Physical Exercise in Patients with Chronic Diseases and Older Adults: An Umbrella Review. Int. J. Environ. Res. Public Health.

[68] Lang S., McLelland C., MacDonald D., Hamilton D.F. (2022). Do digital interventions increase adherence to home exercise rehabilitation? A systematic review of randomised controlled trials. Arch. Physiother..

[69] Albers N., Hizli B., Scheltinga B.L. (2023). l Meijer, E.; Brinkman, W.P. Setting Physical Activity Goals with a Virtual Coach: Vicarious Experiences, Personalization and Acceptance. J. Med. Syst..

